# Supplementary material for: Limited induction of polyfunctional lung-resident memory T cells against SARS-CoV-2 by mRNA vaccination compared to infection
Source: Nat Commun. 2023 Apr 5;14:1887. doi: 10.1038/s41467-023-37559-w (PMC10074357; doi:10.1038/s41467-023-37559-w)
Supplement: Supplementary file 1 — Supplementary Information [file 41467_2023_37559_MOESM1_ESM.pdf]

Supplementary Information

Limited induction of polyfunctional lung-resident memory T cells against SARS-CoV-2 by mRNA vaccination compared to infection

Pieren et al.

Supplementary Figure 1

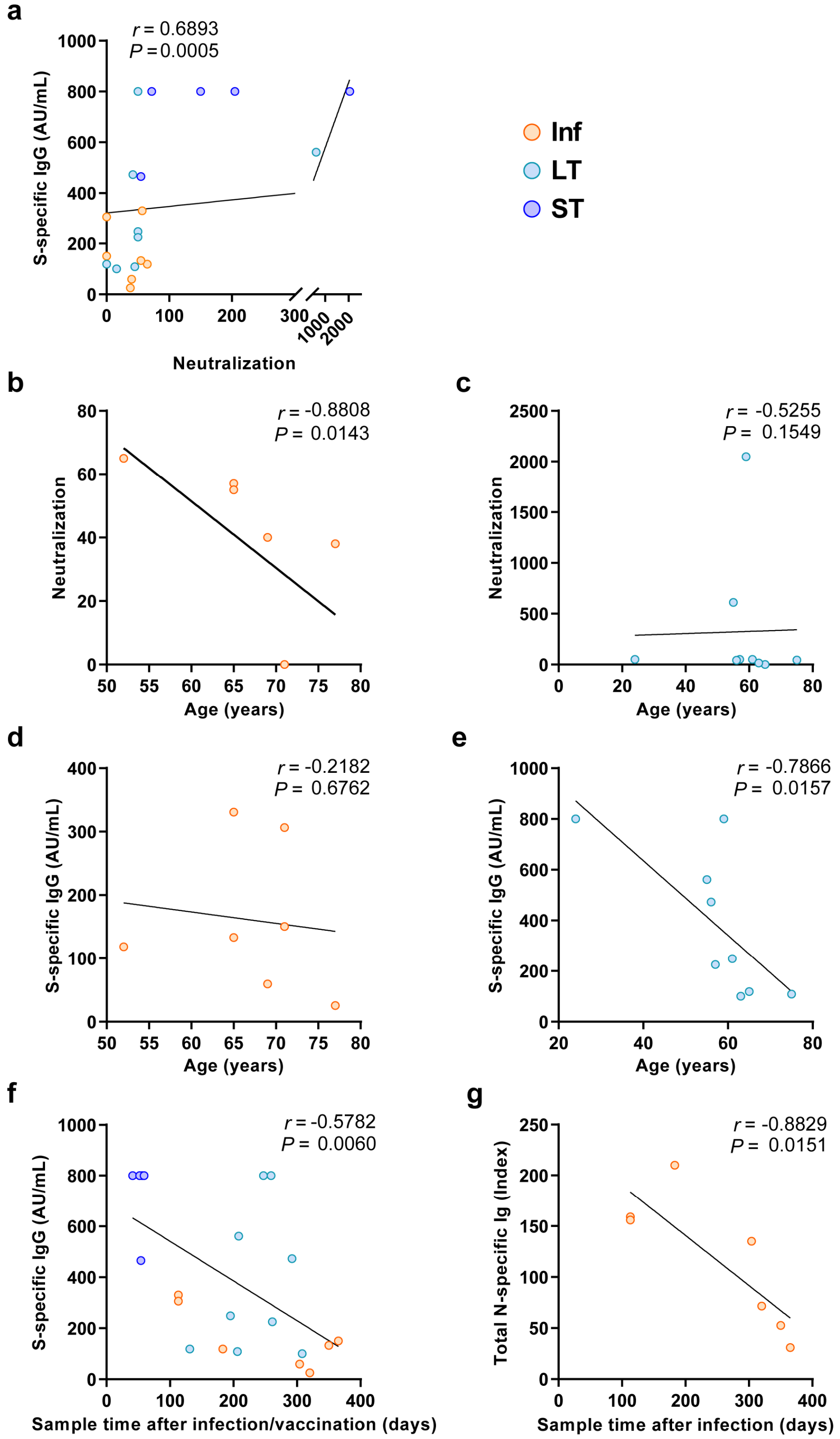

Supplementary Figure 1. Correlation between SARS-CoV-2-specific antibodies, neutralizing capacity, age, and sampling time. Graphs show the relationship between: (**a**) S-specific IgG antibodies (AU/mL) in plasma and SARS-CoV-2 neutralization titer for each group (Inf, convalescent infected, n=7; LT, vaccine 2/3 doses, n=9 and ST, vaccine 3/4 doses, n=5); (**b, c**) SARS-CoV-2 neutralization titer and age of Inf patients (**b**) and LT patients (**c**); (**d, e**) S-specific IgG antibodies (AU/mL) and age of Inf patients (**d**) and LT patients (**e**); (**f**) S-specific IgG antibodies (AU/mL) in all groups and day of sampling after infection or vaccination; and (**g**) N-specific Ig antibodies (index) in Inf patients and day of sampling after infection. Correlations (*r* and *P* values) were assessed by Spearman test (two-sided). Source data are provided as a Source Data file.

Supplementary Figure 2

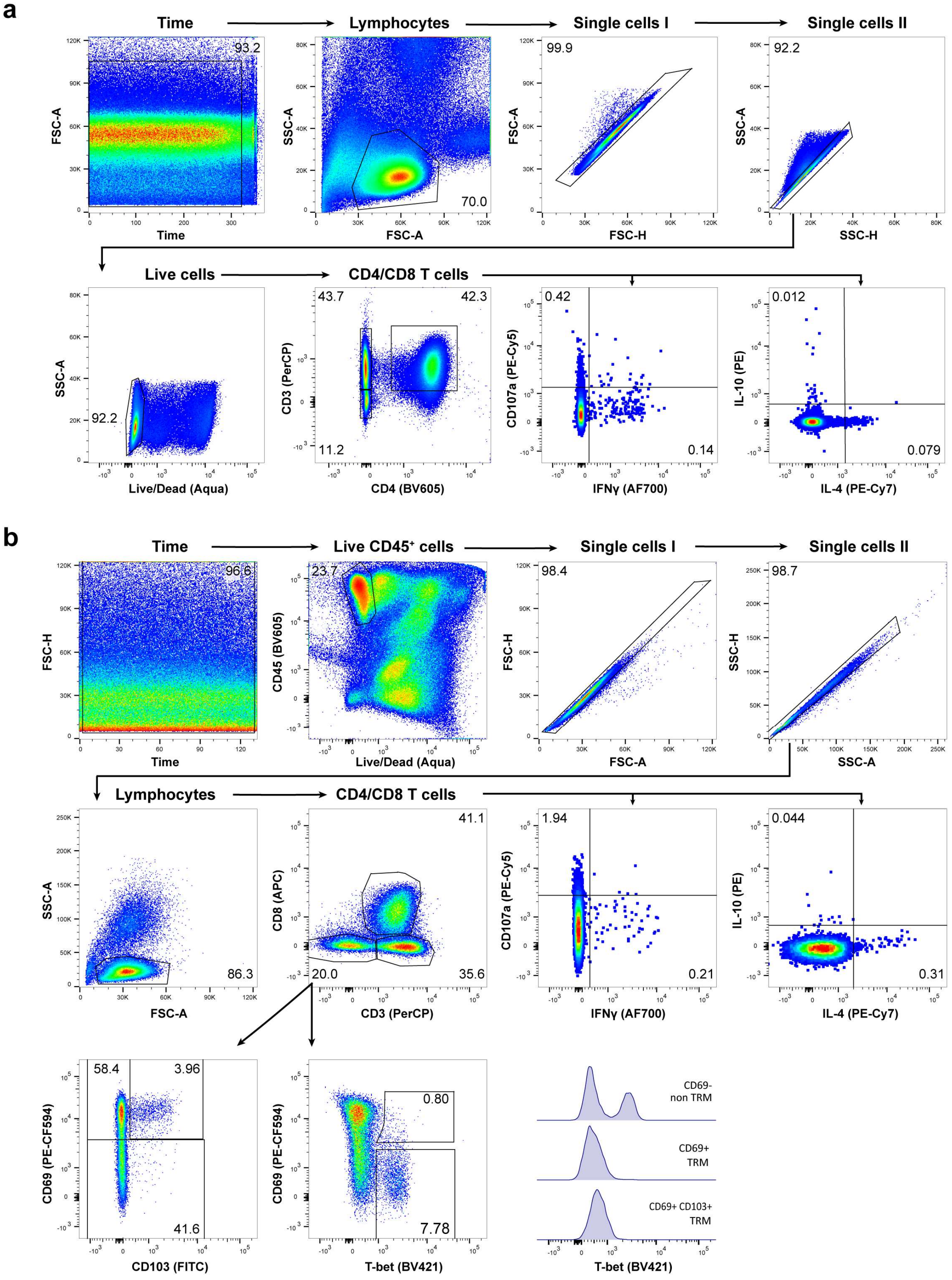

Supplementary Figure 2. Gating strategy for the analysis of T cells present in peripheral blood and lung tissue. (**a, b**) Representative flow-cytometry plots showing the gating strategy towards the identification of $CD4^+$ and $CD8^+$ T cells within PMBC (**a**) and lung tissue (**b**) samples. $CD4^+$ and $CD8^+$ T-cell subsets in PBMCs were identified by gating of time (to exclude disturbances in flow measurements), followed by gating of total lymphocytes, single cells, and live cells. $CD4^+$ and $CD8^+$ T-cell subsets in lung tissue were identified by gating of time, live $CD45^+$ cells, single cells, and lymphocytes.

Supplementary Figure 3

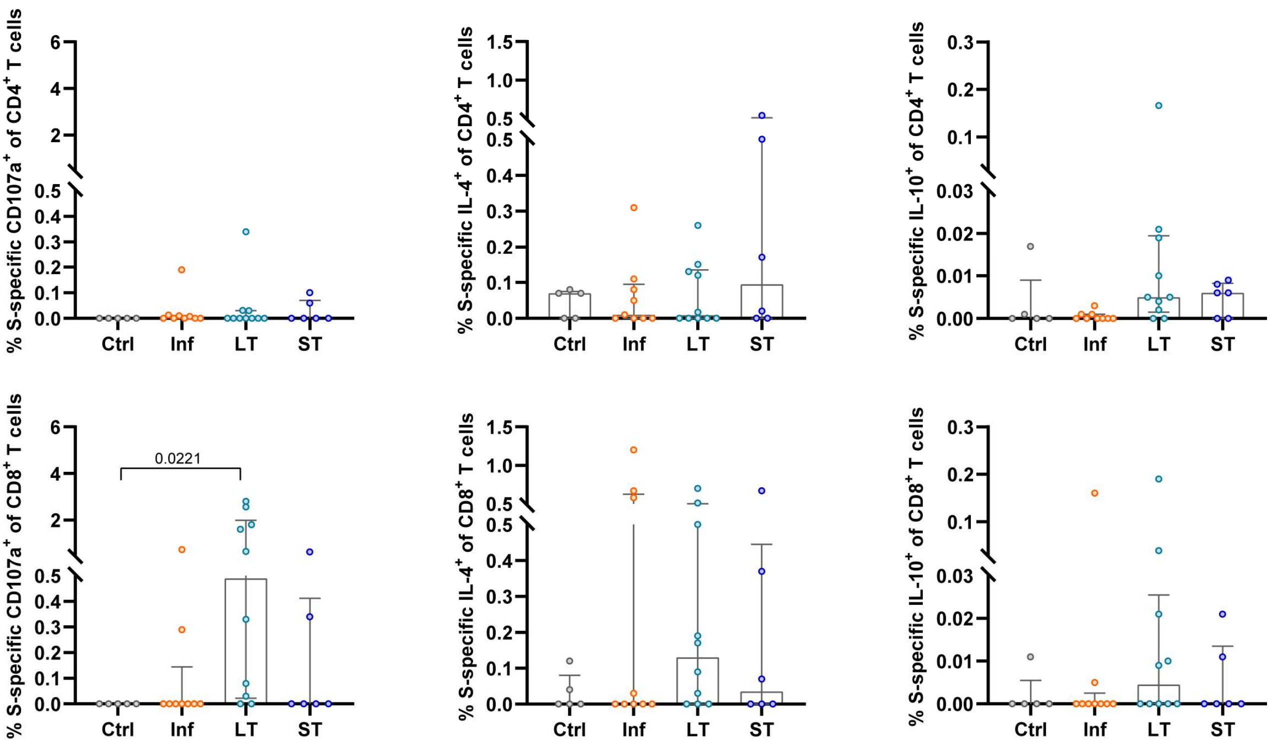

Supplementary Figure 3. Spike-specific T-cell responses (CD107a, IL-4, IL-10) in peripheral blood from convalescent and vaccinated patients. Comparison of the net frequency of CD107a$^+$ (left), IL-4$^+$ (middle), and IL-10$^+$ (right) cells within CD4$^+$ (upper) and CD8$^+$ (lower) T-cell subsets for each of the four groups after exposure of PBMCs to S-peptide pools. Data in bar graphs are shown as median ± IQR, where each dot represents an individual patient for each group (Ctrl, control, n=5; Inf, convalescent infected, n=9; LT, vaccine 2/3doses, n=10 and ST, vaccine 3/4 doses, n=6). Statistical significance was determined by Kruskal-Wallis test (with Dunn's post-test, two-sided). Source data are provided as a Source Data file.

Supplementary Figure 4

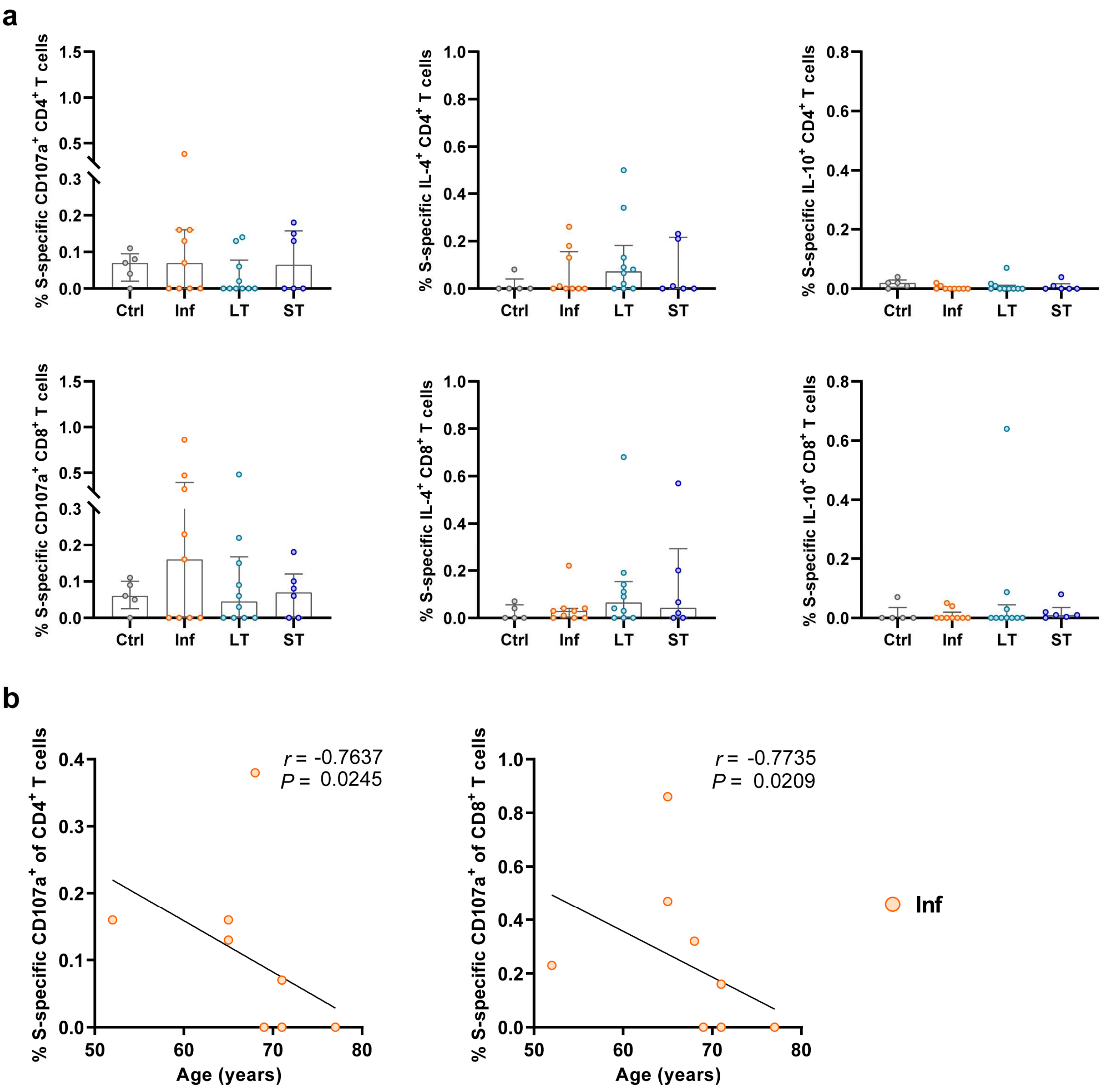

Supplementary Figure 4. SARS-CoV-2-specific T-cell responses (CD107a, IL-4, IL-10) in the lung from convalescent and vaccinated patients. (**a**) Comparison of the net frequency of CD107a^{+} (left), IL-4^{+} (middle), and IL-10^{+} (right) cells within CD4^{+} (upper) and CD8^{+} (lower) T-cell subsets for each of the four groups after exposure of single-cell suspensions of lung tissue to S-peptide pools. Data in bar graphs are shown as median ± IQR, where each dot represents an individual patient for each group (Ctrl, control, n=5; Inf, convalescent infected, n=9; LT, vaccine 2/3 doses, n=10 and ST, vaccine 3/4 doses, n=6).

Statistical significance was determined by Kruskal-Wallis test (with Dunn's post-test, two-sided). (**b**) Correlation between the net frequency of S-specific CD107a$^+$ cells of CD4$^+$ (left) and CD8$^+$ (right) T cells in the lung and age (Inf group). Correlations (*r* and *P* values) were assessed by Spearman test (two-sided). Source data are provided as a Source Data file.

Supplementary Figure 5

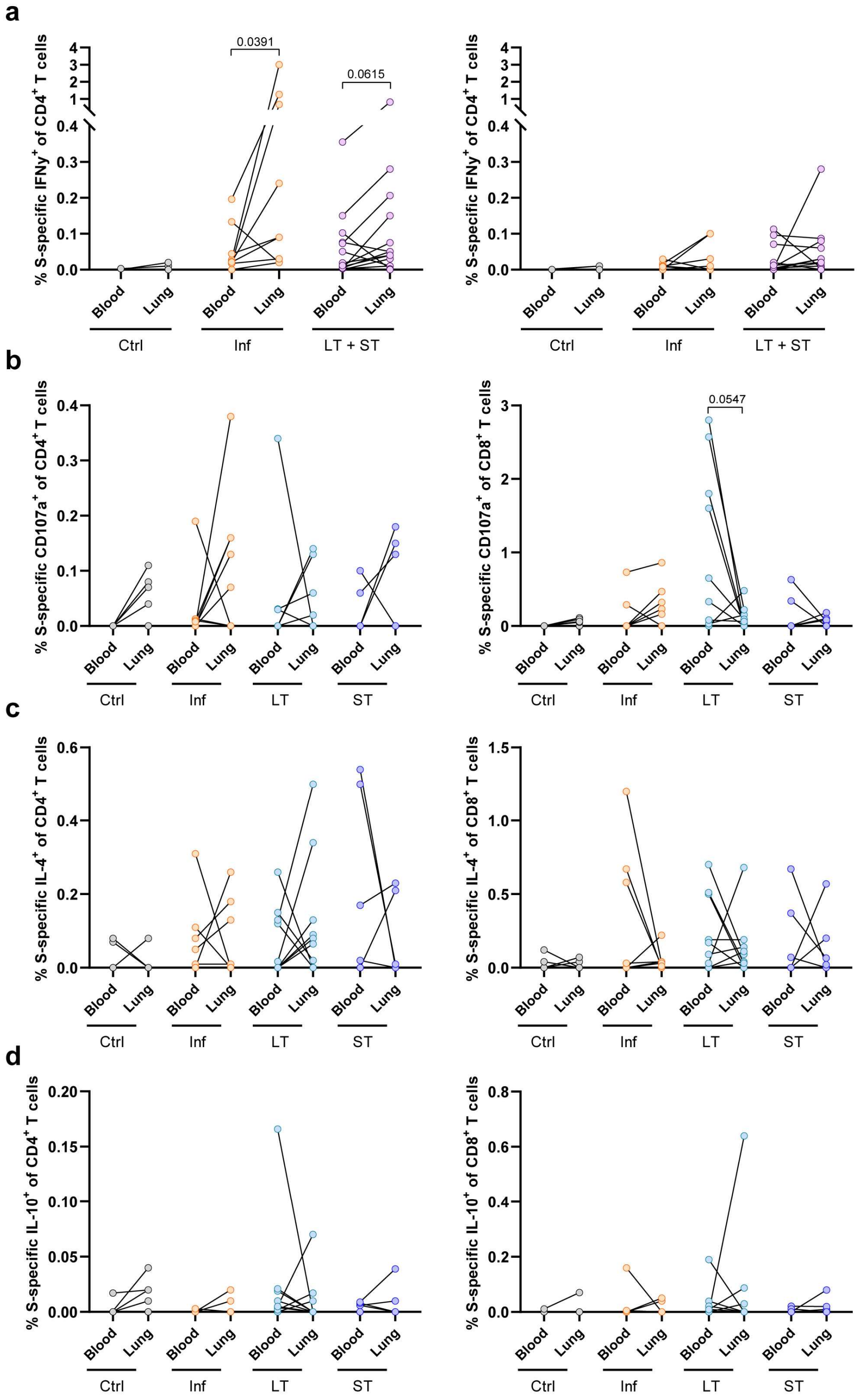

Supplementary Figure 5. Comparison of the frequency of S-peptide specific $CD4^+$ and $CD8^+$ T cells between lung and blood. (**a**) Graphs show the individual patient net frequency of $IFN\gamma^+$ $CD4^+$ and $CD8^+$ T-cell subsets of paired blood and lung samples that were exposed to S-peptide pools with LT and ST vaccinated groups pooled (n=16). **(b-d**) Graphs show the individual patient net frequency of $CD107a^+$ (**b**), $IL\text{-}4^+$ (**c**), and $IL\text{-}10^+$ (**d**) cells within $CD4^+$ (left) and $CD8^+$ (right) T-cell subsets of paired blood and lung samples that were exposed to S-peptide pools (Ctrl, control, n=5; Inf, convalescent infected, n=9; LT, vaccine 2/3 doses, n=10 and ST, vaccine 3/4 doses, n=6). Statistical significance was determined using Friedmann test (with Dunn's post-test, two-sided) for the difference between blood and lung samples within each patient group. Source data are provided as a Source Data file.

Supplementary Figure 6

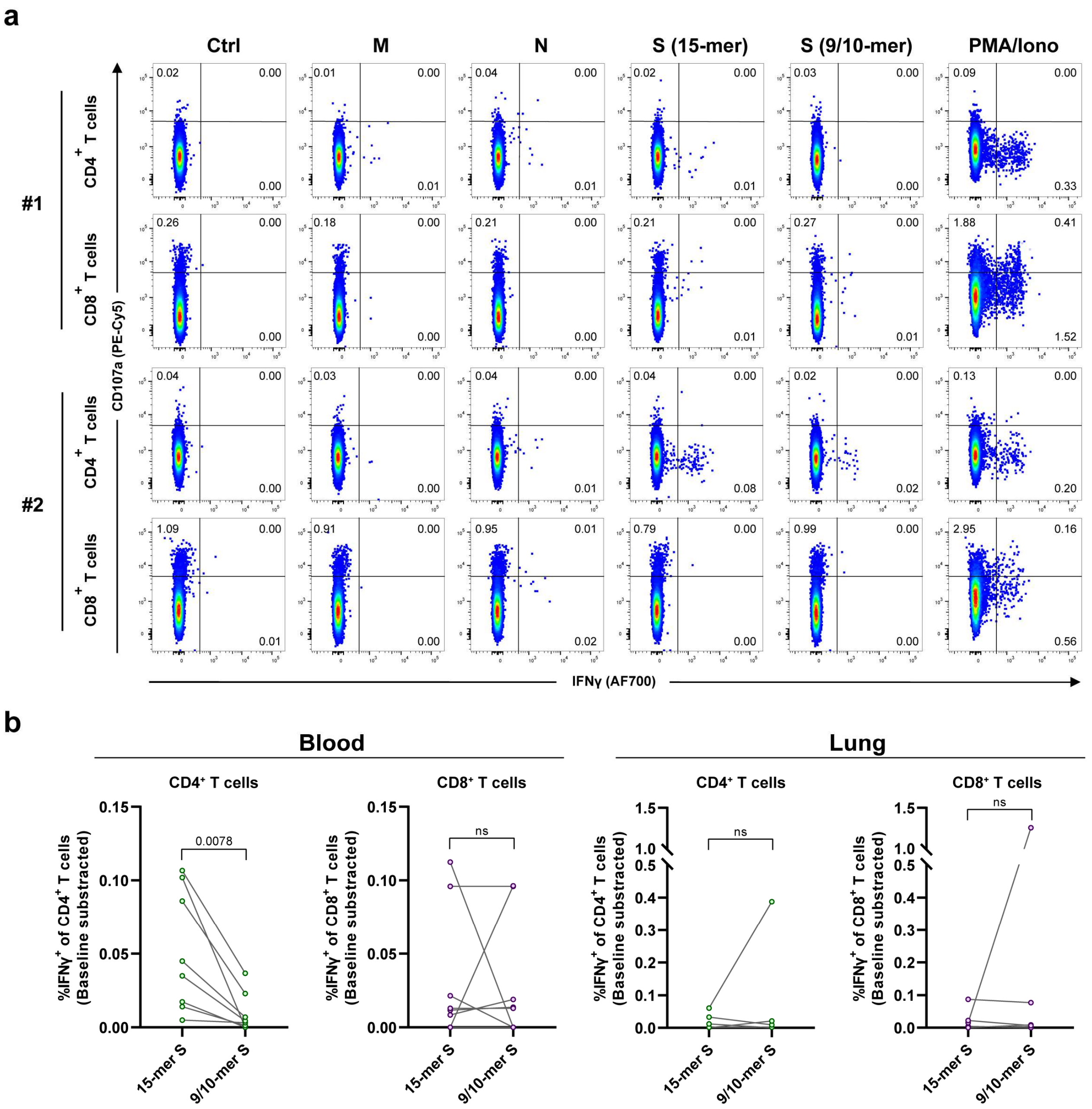

Supplementary Figure 6. Comparison of the T cell response against 15-mer and 9/10-mer S-peptide pools. (**a**) Representative flow-cytometry plots showing $CD4^+$ and $CD8^+$ T cells expressing CD107a and IFNγ of two patients (#1, #2) after exposure of whole PBMCs to M, N, S (either longer 15-mer or shorter 9/10-mer) peptide pools, left unstimulated, or exposed to PMA/ionomycin. (**b**) Comparison of the net frequency (background subtracted) of $IFN\gamma^+$ cells within $CD4^+$ and $CD8^+$ T cell subsets after stimulation of

PBMCs with15-mer or 9/10-mer S-peptide pools. Each pair of dots and connecting line represent the paired response of an individual patient to the two S-peptide pools. Samples of patients included in this comparison were convalescent infected and vaccinated patients (PBMCs n=6, lung n=2), uninfected vaccinated patients (PBMCs n=2, lung n=3). Statistical significance was determined by Wilcoxon test (with Dunn's post-test, two-sided). Source data are provided as a Source Data file.

Supplemental Figure 7

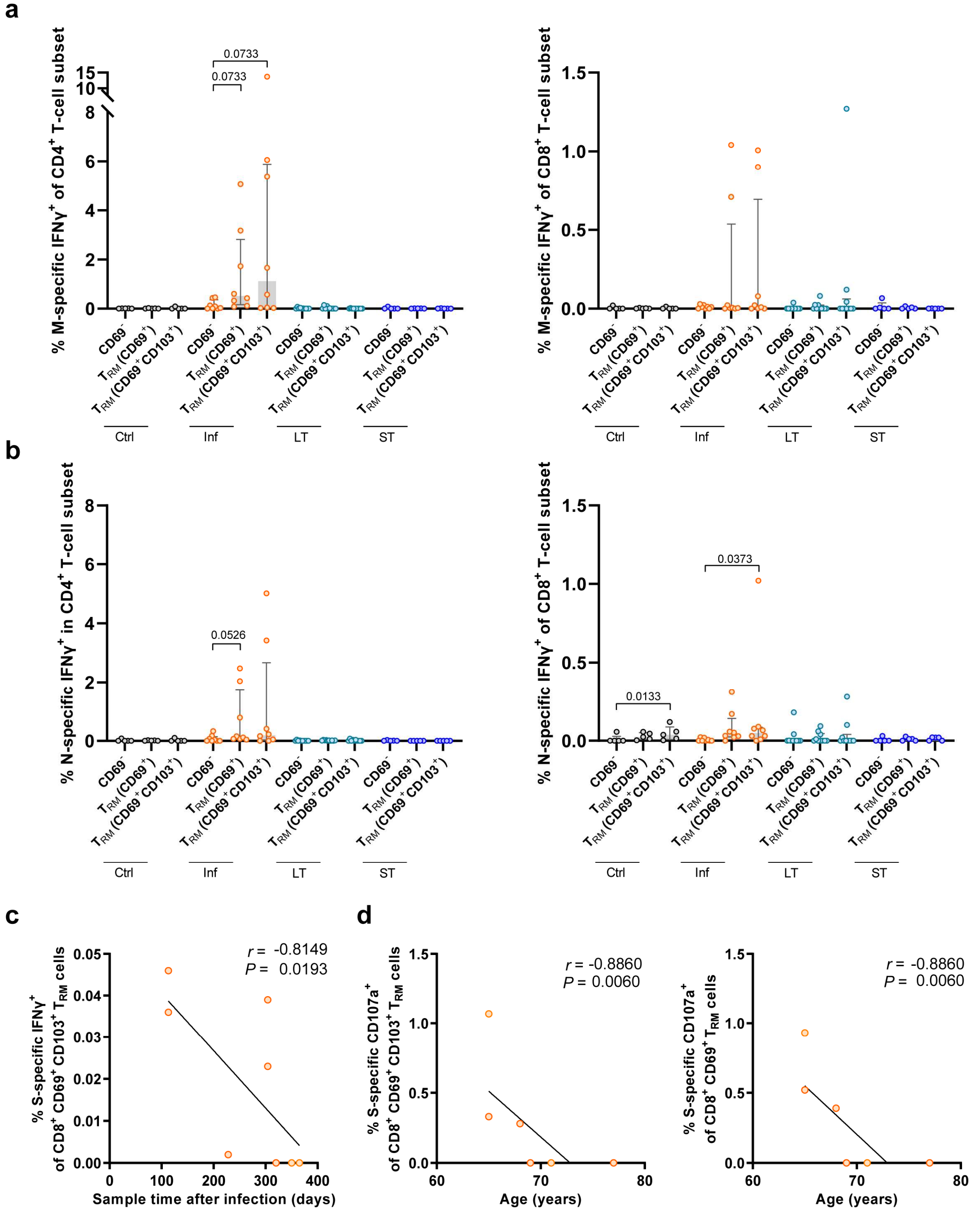

Supplemental Figure 7. The response of CD4$^+$ and CD8$^+$ (non-) T_{RM} cells against M and N peptide pools. (**a, b**) Comparison of the net frequency of IFNγ$^+$ cells within three T_{RM}-cell subsets of CD4$^+$ (left) and CD8$^+$ (right) T cells present in the lung: CD69$^-$ non-T_{RM}, CD69$^+$ T_{RM}, and CD69$^+$CD103$^+$ T_{RM} cells for each group after exposure to (**a**) M- and (**b**) N-peptide pools. Data in bar graphs are shown as median ± IQR, where each dot represents an individual patient for each group (Ctrl, control, n=5; Inf, convalescent infected, n=8; LT, vaccine 2/3 doses, n=10 and ST, vaccine 3/4 doses, n=5). Statistical significance was determined using Friedmann test (with Dunn´s post-test, two-sided) for the difference between the cellular subsets within each patient group. (**c**) Correlation between the net frequency of lung S-specific IFNγ$^+$ cells within the CD8$^+$ CD69$^+$ CD103$^+$ T_{RM} subset and days since confirmed infection and sampling (Inf group). (**d**) Correlation between the net frequency of S-specific CD107a$^+$ CD8$^+$ CD69$^+$ CD103$^+$ T_{RM} cells or CD8$^+$ CD69$^+$ T_{RM} cells and age (Inf group). Correlations (*r* and *P* values) were assessed by Spearman test (two-sided). Source data are provided as a Source Data file.

Supplementary Figure 8

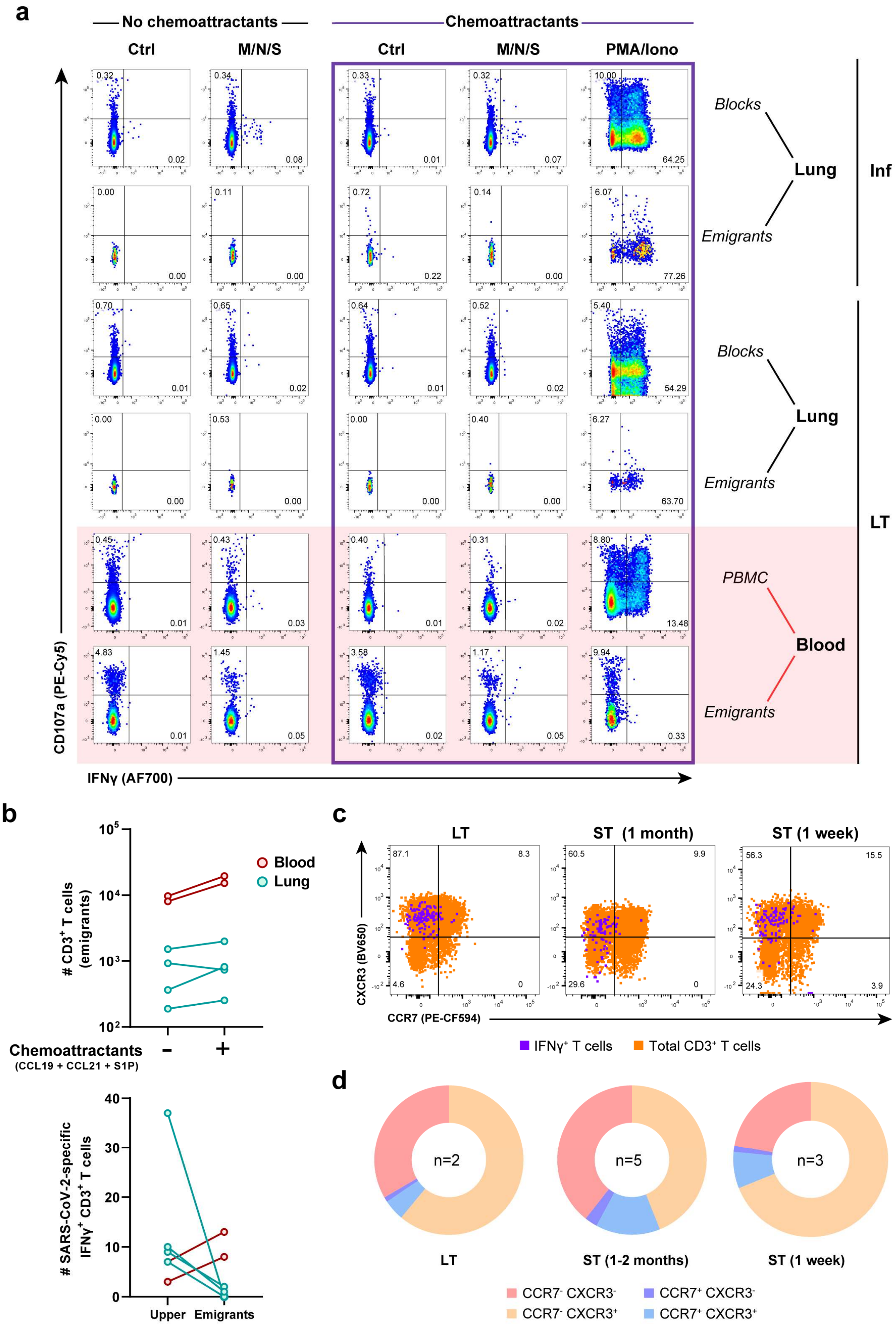

Supplementary Figure 8. Homing and residency of SARS-CoV-2 specific T cells. (**a**) Representative flow-cytometry plots showing $CD3^+$ T cells from the lung expressing CD107a and IFNγ of one convalescent infected vaccinated patient (Inf, upper two rows) and one uninfected vaccinated patient (LT, middle two rows) and from blood (lower two rows), with and without exposure to CCL19, CCL21 and S1P in a transwell and subsequent stimulation with M, N, and S-peptide pools, left unstimulated, or PMA/ionomycin. (**b**) Graphs show the number of $CD3^+$ T cells emigrated with and without chemoattractant conditions (upper graph) and the number of SARS-CoV-2-specific $IFN\gamma^+$ $CD3^+$ T cells that remained in the upper transwell and those that emigrated (lower graph) in both lung (blue) and blood (red) samples. (**c**) Representative flow-cytometry plots showing S-specific $IFN\gamma^+$ $CD4^+$ T cells (in purple) among the distribution of CXCR3 and CCR7 in the total live T cell subset (orange) of one LT patient (left), one ST patient (middle), and one convalescent infected and recently vaccinated patient (ST-1 week; right). (**d**) Donut charts showing the proportion of S-specific $IFN\gamma^+$ $CD4^+$ T cells based on CCR7 and CXCR3 expression of LT patients (n=2), ST patients (n=5), and convalescent infected and recently vaccinated patients (n=3). Source data are provided as a Source Data file.

Supplementary Figure 9

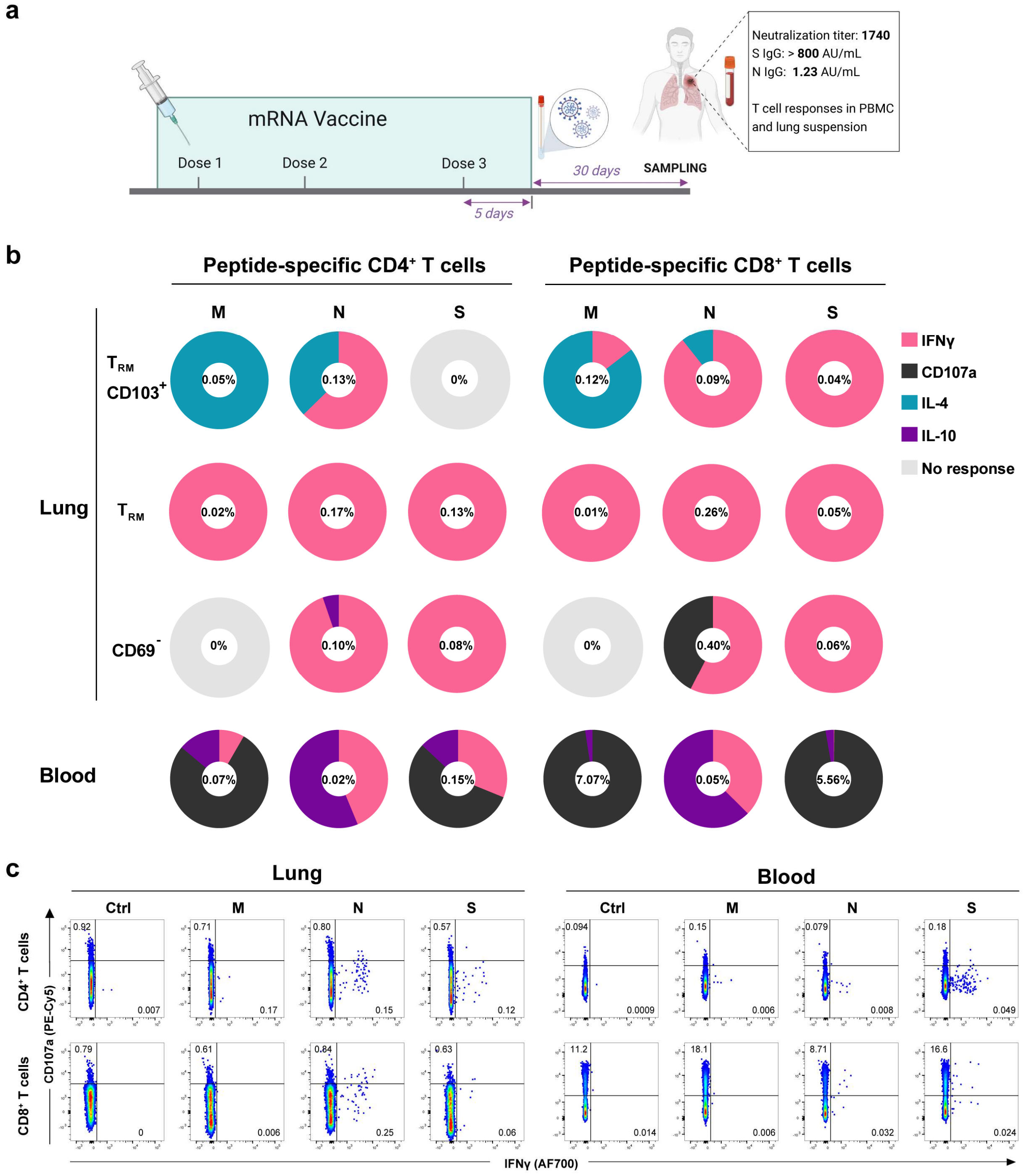

Supplementary Figure 9. T-cell responses for patient #174. (**a**) Timeline for patient 174, indicating vaccination against SARS-CoV-2, infection with SARS-CoV-2, and acquisition of blood and lung samples. (**b**) Donut charts displaying the net contribution of each functional marker (IFNγ, CD107a, IL-4, IL-10, or no response) to the M-, N-, and S-specific $CD4^+$ and $CD8^+$ T-cell response within the lung resident and non-resident T-cell subsets and in peripheral blood for patient 174. The frequency shown inside each donut chart represents the accumulated mean response of all functions. (**c**) Flow-cytometry plots of patient 174 showing $CD4^+$ (upper) and $CD8^+$ (lower) T cells expressing CD107a and IFNγ after exposure of lung single-cell suspensions (left) and PBMCs (right) to M-, N- and S-peptide pools or left unstimulated. Source data are provided as a Source Data file.

Supplemental Figure 10

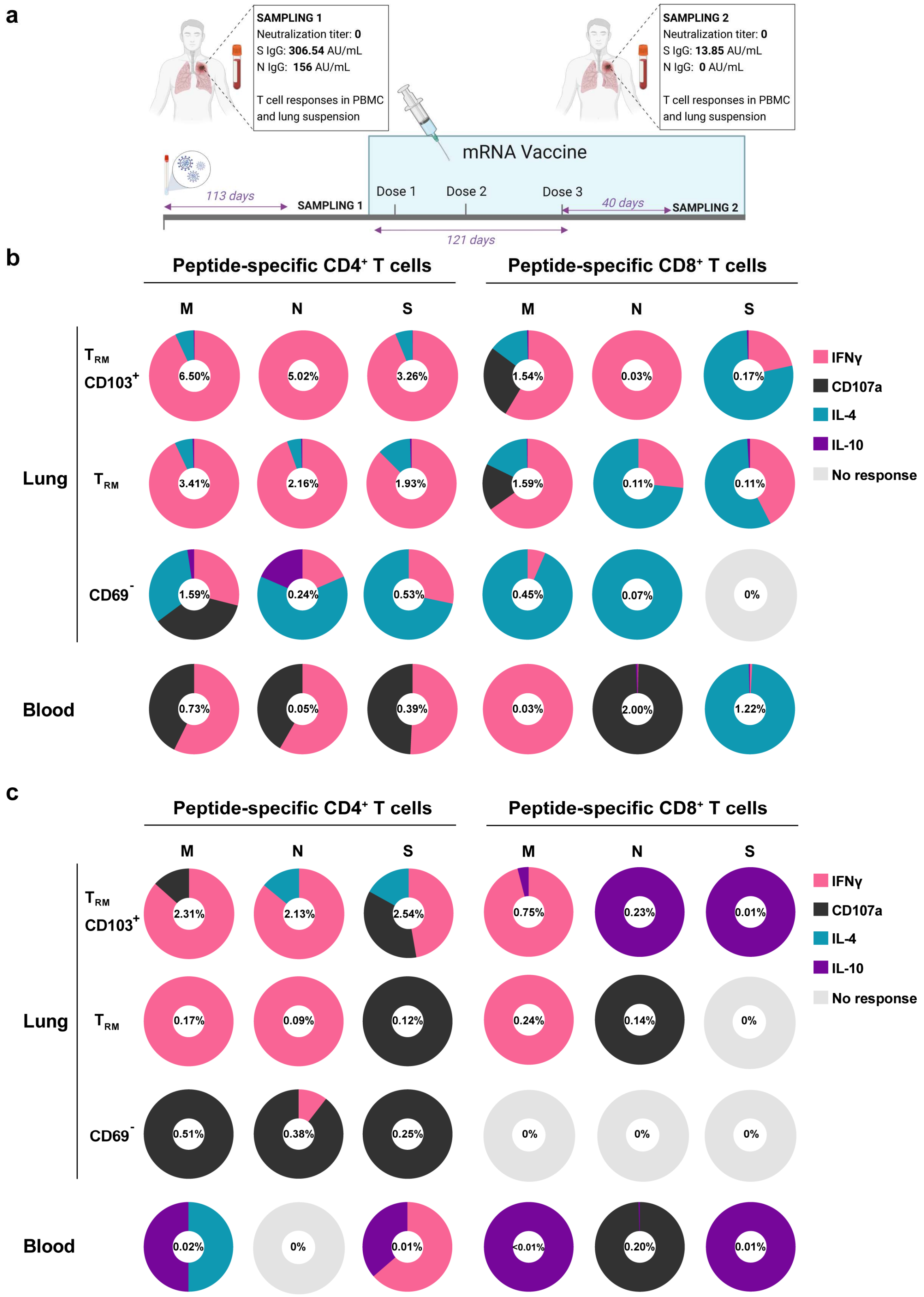

Supplementary Figure 10. T-cell responses for patient #162, longitudinal samples. (**a**) Timeline for patient 162 who was longitudinally sampled, first ~4 months after SARS-CoV-2 infection and then ~1 month after third dose mRNA-vaccination. (**b, c**) Donut charts displaying the net contribution of each functional marker (IFNγ, CD107a, IL-4, IL-10, or no response) to the M-, N-, and S-specific $CD4^+$ and $CD8^+$ T-cell response within the lung resident and non-resident T-cell subsets and in peripheral blood for patient 162 in the first sample (**b**) and second sample (**c**). The frequency shown inside the donut chart represents the accumulated mean response of all functions. Source data are provided as a Source Data file.

Supplementary Figure 11

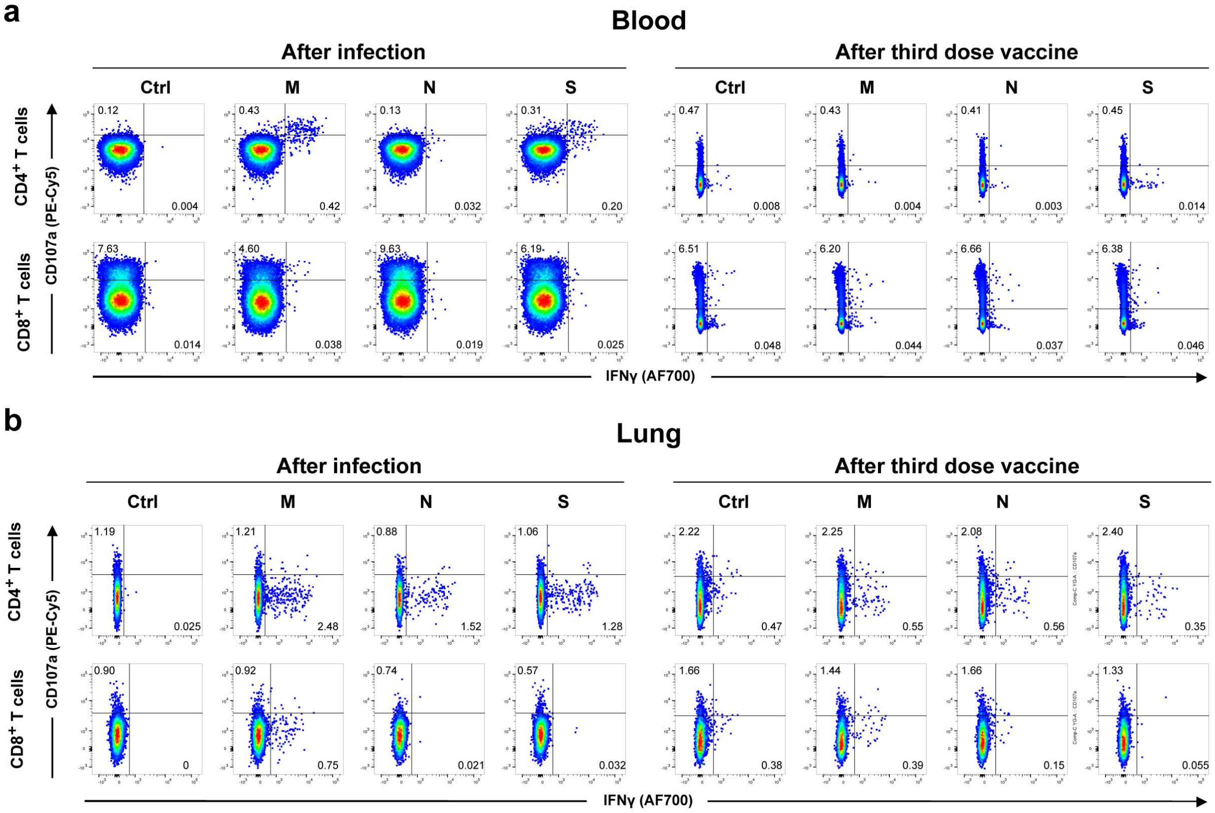

Supplementary Figure 11. Longitudinal patterns of SARS-CoV-2-specific T-cell responses in lung and blood for patient #162. (**a, b**) Flow-cytometry plots showing longitudinal data (left, after infection, sample 1; right, after third-dose vaccination, sample 2) of patient 162 showing CD4$^+$ (top) and CD8$^+$ (bottom) T cells expressing CD107a and IFNγ after exposure of PBMCs (**a**) and lung cell suspension (**b**) to M-, N-, and S-peptide pools or left unstimulated (Ctrl).

Supplementary Table 1. Patient characteristics

	Control n=5	Convalescent infected n=9‡	Long-term vaccinated n=10‡	Short-term vaccinated n=6‡	*P* value between groups
Age (years), median [min.-max.]	**67** [63-74]	**69** [52-77]	**60** [24-79]	**73** [43-81]	0.1529[a]
Female, n (%)	**1/5** (20%)	**1/9** (11%)	**5/10** (50%)	**4/6** (67%)	0.1014[b]
Days after infection* or vaccination, median [IQR]	N/A	**304** [183-320]	**228** [198-261]	**53** [45-56]	**0.0011[a]**
Spike-specific IgG** (AU/mL), median [IQR] (AU/mL)	**<1.85** [1.85-1.85]	**133.1** [89.0-228.5]	**248.5** [118.9-561.5]	**800** [800-800]	**0.0005[a]**
Total nucleocapsid-specific Ig** (Index), median [IQR]	**0.07** [0.07-0.08]	**135** [62.1-157.5]	**0.09** [0.07-0.12]	**0.09** [0.06-0.09]	**0.0014[a]**
Virus neutralization titer***, median [IQR]	**0** [0-0]	**40** [19-56]	**50** [42-50]	**150** [72-205]	**0.0393[a]**
Patients with SARS-CoV-2 neutralization capacity, n (%)	**0/5** (0%)	**5/7** (71%)	**8/9** (89%)	**5/5** (100%)	0.4877[b]
Diagnosis of lung cancer after surgery, n (%)	**5/5** (100%)	**9/9** (100%)	**7/10 (**70%)	**5/6** (83%)	0.2022[b]
Cancer treatment at time of surgery, n (%)	**0/5** (0%)	**0/9** (0%)	**0/10** (0%)	**1/6** (17%)	0.2470[b]

* Confirmed by RT-PCR for SARS-CoV-2

** Measured by anti-SARS-CoV-2 S and N immunoassay

*** Measured by SARS-CoV-2 neutralization assay

‡ Plasma samples were not available for every patient

N/A not available

[a] Kruskal-Wallis test with Dunn's post-test

[b] Chi-square test
