# Supplementary material for: Limited induction of polyfunctional lung-resident memory T cells against SARS-CoV-2 by mRNA vaccination compared to infection
Source: Nat Commun. 2023 Apr 5;14:1887. doi: 10.1038/s41467-023-37559-w (PMC10074357; doi:10.1038/s41467-023-37559-w)
Supplement: Supplementary file 3 — Reporting Summary [file 41467_2023_37559_MOESM3_ESM.pdf]

nature portfolio

Corresponding author(s): Meritxell Genescà Ferrer

Last updated by author(s): Mar 9, 2023

Reporting Summary

Nature Portfolio wishes to improve the reproducibility of the work that we publish. This form provides structure for consistency and transparency in reporting. For further information on Nature Portfolio policies, see our Editorial Policies and the Editorial Policy Checklist.

Statistics

For all statistical analyses, confirm that the following items are present in the figure legend, table legend, main text, or Methods section.

n/a	Confirmed	
☐	☒	The exact sample size (*n*) for each experimental group/condition, given as a discrete number and unit of measurement
☐	☒	A statement on whether measurements were taken from distinct samples or whether the same sample was measured repeatedly
☐	☒	The statistical test(s) used AND whether they are one- or two-sided<br>*Only common tests should be described solely by name; describe more complex techniques in the Methods section.*
☐	☒	A description of all covariates tested
☐	☒	A description of any assumptions or corrections, such as tests of normality and adjustment for multiple comparisons
☐	☒	A full description of the statistical parameters including central tendency (e.g. means) or other basic estimates (e.g. regression coefficient) AND variation (e.g. standard deviation) or associated estimates of uncertainty (e.g. confidence intervals)
☐	☒	For null hypothesis testing, the test statistic (e.g. *F*, *t*, *r*) with confidence intervals, effect sizes, degrees of freedom and *P* value noted<br>*Give P values as exact values whenever suitable.*
☒	☐	For Bayesian analysis, information on the choice of priors and Markov chain Monte Carlo settings
☒	☐	For hierarchical and complex designs, identification of the appropriate level for tests and full reporting of outcomes
☒	☐	Estimates of effect sizes (e.g. Cohen's *d*, Pearson's *r*), indicating how they were calculated

Our web collection on statistics for biologists contains articles on many of the points above.

Software and code

Policy information about availability of computer code

Data collection	BD FACSDiva v8.0
Data analysis	GraphPad Prism (version 8.3.0) for statistical analysis and FlowJo v10.7.1 (TreeStar) software for flow cytometry.

For manuscripts utilizing custom algorithms or software that are central to the research but not yet described in published literature, software must be made available to editors and reviewers. We strongly encourage code deposition in a community repository (e.g. GitHub). See the Nature Portfolio guidelines for submitting code & software for further information.

Data

Policy information about availability of data

All manuscripts must include a data availability statement. This statement should provide the following information, where applicable:

- Accession codes, unique identifiers, or web links for publicly available datasets
- A description of any restrictions on data availability
- For clinical datasets or third party data, please ensure that the statement adheres to our policy

The data supporting the findings of this study are available in the main article and its supplementary files. Source data are provided with this paper.

Human research participants

Policy information about studies involving human research participants and Sex and Gender in Research.

Reporting on sex and gender	We indirectly used the term sex when describing the patient characteristics of our cohort by including the number and percentage of female patients in each patient group. Individuals included in our study were patients at the Vall d´Hebron Hospital and their sex was derived based on the clinical data available and how patients were registered in the national health system. No selection based on sex or gender was applied.
Population characteristics	Patients undergoing lung resection for various reasons at the Vall d'Hebron University Hospital were recruited through the Thoracic Surgery Service and invited to participate. Adult patients included in this study were 24 years old or older and were assigned to one of the following groups: I.) SARS-CoV-2 uninfected unvaccinated individuals (Ctrl, n=5), II.) unvaccinated long-term SARS-CoV-2 convalescent individuals (Inf, n=9, convalescent for ~4-12 months), III.) uninfected and long-term two- or three-dose vaccinated individuals (LT, n=10, ~4-10 months after the last vaccine), and IV.) uninfected and short-term three- or four-dose vaccinated individuals (ST, n=6, 1.3-1.8 months after the last vaccine). Patient information is summarized in Supplementary Table 1 and a schematic summary is shown in Figure 1a.<br>To assign patients to their group, clinical history was consulted to confirm or rule out previous infection with SARS-CoV-2. Moreover, plasma samples of most patients were analysed for the presence of total Ig against N-protein and IgG against S-protein, which discriminated Control patients (negative for N and S IgG), Convalescent Infection patients (positive for N and S) and vaccinated uninfected patients (negative for N and positive for S).
Recruitment	Recruitment of patients relied on their need to undergo lung resection for various reasons. Thus, recruitment and availability of patients primarily relied on surgical planification. Additionally, recruitment of patients to our predefined study groups was dependent on their vaccination and/or SARS-CoV-2 infection status as described in the Methods section. Surgeries were performed at the Vall d'Hebron University Hospital and patients were recruited through the Thoracic Surgery Service and invited to participate. It is unlikely that this selection created a bias in our results as we predefined group conditions and included patients meeting these requirements. As additional controls for certain analyses requiring only blood, healthy volunteers recently boosted with a fourth vaccine dose (within 1 week) were recruited. Lastly, no other selection criteria were applied to all groups present in this study.
Ethics oversight	This study was performed in accordance with the Declaration of Helsinki and approved by the corresponding Institutional Review Board (PR(AG)212/2020) of the Vall d'Hebron University Hospital (HUVH), Barcelona, Spain. Written informed consent was provided by all patients recruited to this study.

Note that full information on the approval of the study protocol must also be provided in the manuscript.

Field-specific reporting

Please select the one below that is the best fit for your research. If you are not sure, read the appropriate sections before making your selection.

☒ Life sciences ☐ Behavioural & social sciences ☐ Ecological, evolutionary & environmental sciences

For a reference copy of the document with all sections, see nature.com/documents/nr-reporting-summary-flat.pdf

Life sciences study design

All studies must disclose on these points even when the disclosure is negative.

Sample size	No sample-size calculations were performed. Next to tissue availability, sample size was determined to be adequate based on the magnitude and consistency of measurable differences between groups.
Data exclusions	No data were excluded after selection of patients meeting the requirements for assignment to one of the four study groups. Study groups were: I.) SARS-CoV-2 uninfected unvaccinated individuals, II.) unvaccinated long-term SARS-CoV-2 convalescent individuals (convalescent for ~4-12 months), III.) uninfected and long-term two- or three-dose vaccinated individuals (~4-10 months after the last vaccine), and IV.) uninfected and short-term three- or four-dose vaccinated individuals (1.3-1.8 months after the last vaccine).
Replication	Given the uniqueness of the paired blood and lung samples, experiments to measure SARS-CoV-2 specific responses in blood and lung samples were optimized and replicated before starting this study. Individual lung and blood samples were not replicated due to sample limitation.<br>After optimization, transwell experiments were performed with available blood and lung samples. A minimum of nine Transwell replicates were used for each experiment for blood and lung samples separately. All replication attempts were successful.
Randomization	Allocation to experimental groups was not random, as our research aim did not allow for random allocation. Patients were first selected on meeting predefined criteria of our study groups based on their recorded clinical history (i.e. history of proven SARS-CoV-2 infection and mRNA vaccination status). Predefined study group criteria were: I.) SARS-CoV-2 uninfected unvaccinated individuals, II.) unvaccinated long-term SARS-CoV-2 convalescent individuals (convalescent for ~4-12 months), III.) uninfected and long-term two- or three-dose vaccinated individuals (~4-10 months after the last vaccine), and IV.) uninfected and short-term three- or four-dose vaccinated individuals (1.3-1.8 months after the last vaccine). After the first selection based on clinical records, the status of patients in the vaccine groups was confirmed by serological testing: vaccinated patients with presence of total Ig antibodies against the N protein were excluded from the study.

Blinding

Investigators were not blinded during this study. Because of the uniqueness of the paired blood and lung samples and the criteria of our groups this was not possible.

Reporting for specific materials, systems and methods

We require information from authors about some types of materials, experimental systems and methods used in many studies. Here, indicate whether each material, system or method listed is relevant to your study. If you are not sure if a list item applies to your research, read the appropriate section before selecting a response.

Materials & experimental systems

n/a	Involved in the study
	x Antibodies
	x Eukaryotic cell lines
x	Palaeontology and archaeology
x	Animals and other organisms
x	Clinical data
x	Dual use research of concern

Methods

n/a	Involved in the study
x	ChIP-seq
	x Flow cytometry
x	MRI-based neuroimaging

Antibodies

Antibodies used

anti-CD107a (PE-Cy5, clone H4A3, BD Biosciences, #555802; 1:20)
anti-CD103 (FITC, clone Ber-ACT8, Biolegend, #350204; 1:50)
anti-CD69 (PE-CF594, clone FN50, BD Biosciences, #562617; 1:30)
anti-CD40 (APC-Cy7, clone HB14, Biolegend, #313017; 1:10)
anti-CD8 (APC, clone RPA-T8, BD Biosciences, #561952; 1:50)
anti-CD3 (BV650, clone UCHT1, BD Biosciences, #563851; 1:166)
anti-CD45 (BV605, clone HI30, BD Biosciences, #564047; 1:50)
anti-IL-4 (PE-Cy7, clone 8D4-8, eBioscience, #25-7049-82; 1:40)
anti-IL-10 (PE, clone JES3-19F1, BD Biosciences, #559330; 1:10)
anti-T-bet (BV421, clone 4B10, Biolegend, #644815; 1:40)
anti-IFNγ (AF700, clone B27, Invitrogen, #MHCIFG29; 1:40)
anti-CCR7 (PE-CF594, clone 150503, BD Biosciences, #562381; 1:100)
anti-CXCR3 (BV650, clone G025H7, BD Biosciences, #353730; 1:28)
anti-CD3 (PerCP, clone SK7, BD Biosciences, #340663; 1:10)
anti-CD4 (BV605, clone RPA-T4, BD Biosciences, #562658; 1:20)
anti-CD56 (FITC, clone B159, BD Biosciences, #562794; 1:50)
anti-Caspase-3 (AF647, clone C92-605, BD Biosciences, #560626; 1:33)
anti-Bcl-2 (BV421, clone 100, Biolegend, #658709; 1:80)

Validation

All antibodies used in this study are commercially available and were commercially validated. They have been used according to manufacturer data sheets available at the manufacturer´s website. In house titration of antibodies was performed to determine the optimal dilution for our experiments.

According to the manufacturer's website, anti-CD107a (clone H4A3) antibody (BD Biosciences, Cat#555802) has been routinely tested on the fixed and permeabilized Jurkat cells by flow cytometry with Cytofix/Cytoperm (Cat. No. 554714) for fixation and permeabilization. This antibody was previously validated in Chen JW et al., 1988, J Biol Chem.
According to the manufacturer's website, anti-CD103 (clone Ber-ACT8) antibody (Biolegend, Cat# 350204) is quality control tested by immunofluorescent staining with flow cytometric analysis. Flow cytometric analysis of CD103 expression in human peripheral blood mononuclear cells is provided on the website.
According to the manufacturer's website, anti-CD69 (clone FN50) antibody (BD Biosciences, Cat#562617) is suitable for flow cytometry. Flow cytometric analysis of CD69 expression by stimulated peripheral blood mononuclear cells is provided on the website.
According to the manufacturer's website, anti-CD40 (clone HB14) antibody (Biolegend, Cat#313017) is quality control tested by immunofluorescent staining with flow cytometric analysis. Flow cytometric analysis of CD40 expression in human peripheral blood lymphocytes is provided on the website.
According to the manufacturer's website, anti-CD8 (clone RPA-T8) antibody (BD Biosciences, Cat#561952) is routinely tested for flow cytometry. Flow cytometric analysis of CD8 expression on human peripheral blood lymphocytes is provided on the website.
According to the manufacturer's website, anti-CD3 (clone UCHT1) antibody (BD Biosciences, Cat#563851) is routinely tested for flow cytometry. Flow cytometric analysis of CD3 expression on human peripheral blood lymphocytes is provided on the website.
According to the manufacturer's website, anti-CD45 (clone HI30) antibody (BD Biosciences, Cat#564047) is routinely tested for flow cytometry. Flow cytometric analysis of CD45 expression on human peripheral blood lymphocytes is provided on the website.
According to the manufacturer's website, anti-IL-4 (clone 8D4-8) antibody (eBioscience, Cat# 25-7049-82) is suitable for flow cytometry. Flow cytometric analysis of IL-4 expression on human peripheral cells is provided on the website.
According to the manufacturer's website, anti-IL-10 (clone JES3-19F1) antibody (BD Biosciences, Cat# 559330) has been routinely

tested on fixed and permeabilized human peripheral blood lymphocytes by flow cytometry. Flow cytometric analysis of IL-10 expression by stimulated CD14+ human monocytes is provided on the website.
According to the manufacturer's website, anti-IFNg (clone B27) antibody (Invitrogen, Cat# MHCIFG29) is suitable for flow cytometry. This antibody was previosly validated by Demers KR et al., 2016, PLos pathogens.
According to the manufacturer's website, anti-T-bet (clone 4B10) antibody (Biolegend, Cat# 644815) is quality tested for intracellular immunofluorescent staining with flow cytometric analysis. Flow cytometric analysis of T-bet expression on human peripheral blood lymphocytes is provided on the website.
According to the manufacturer's website, anti-CD197 (CCR7, clone 150503) antibody (BD Biosciences, Cat# 562381) is routinely tested for flow cytometry. Flow cytometric analysis of CD197 (CCR7) expression on human peripheral blood lymphocytes is provided on the website.
According to the manufacturer's website, anti-CD186 (CXCR3, clone G025H7) antibody (Biolegend, Cat# 353730) is quality tested by immunofluorescent staining with flow cytometric analysis. This antibody was previously validated in Kenderes KJ, et al., 2018, Cell Rep.
According to the manufacturer's website, anti-CD3 (clone SK7) antibody (BD Biosciences, Cat# 562658) is intended for in vitro diagnostic use in the identification of cells expressing the CD3 antigen, using a BD FACS™ brand flow cytometer. This antibody was previously validated in van Dongen JJM et al., 1988, Blood.
According to the manufacturer's website, anti-CD4 (clone RPA-T4) antibody (BD Biosciences, Cat# 562658) is suitable for flow cytometry. Flow cytometric analysis of CD4 expression on human peripheral blood lymphocytes is provided on the website.
According to the manufacturer's website, anti-CD56 (clone B159) antibody (BD Biosciences, Cat# 562794) is suitable for flow cytometry. Flow cytometric analysis of CD56 expression on human peripheral lymphocytes is provided on the website.
According to the manufacturer's website, anti-Caspase-3 (clone C92-605) antibody (BD Biosciences, Cat# 560626) is suitable for intracellular staining in flow cytometry. Flow cytometric analysis of apoptotic and non-apoptotic populations for active caspase-3 in Jurkat cells is provided on the website.
According to the manufacturer's website, anti-Bcl-2 (clone 100) antibody (Biolegend, Cat# 658709) is suitable for intracellular immunofluorescent staining with flow cytometric analysis. Flow cytometric analysis of Bcl-2 expression on human peripheral blood lymphocytes is provided on the website.

Eukaryotic cell lines

Policy information about cell lines and Sex and Gender in Research

Cell line source(s)
HEK293T (ATCC)
VERO (ATCC CRL-1587)

Authentication
None of the cell lines used were authenticated

Mycoplasma contamination
Cell lines used were not tested for mycoplasma contamination

Commonly misidentified lines (See ICLAC register)
No commonly misidentified cell lines were used.

Flow Cytometry

Plots

Confirm that:

- [x] The axis labels state the marker and fluorochrome used (e.g. CD4-FITC).
- [x] The axis scales are clearly visible. Include numbers along axes only for bottom left plot of group (a 'group' is an analysis of identical markers).
- [x] All plots are contour plots with outliers or pseudocolor plots.
- [x] A numerical value for number of cells or percentage (with statistics) is provided.

Methodology

Sample preparation
For all participants, whole blood was collected with EDTA anticoagulant. Plasma was collected and stored at −80°C (except for 4 patients distributed among the different groups, as indicated in Supplementary Table 1, for which this sample was not available) and PBMCs were isolated via Ficoll–Paque separation and processed immediately for stimulation assays.
Freshly isolated PBMCs were labeled as indicated in materials and methods section.
For lung biopsies, immediately following surgery, healthy areas from patients undergoing lung resection were collected in antibiotic-containing RPMI 1640 medium and processed and labeled as described in the materials and methods section.

Instrument
BD FACS Fortessa analyzer.

Software
FACS Diva for data collection and FlowJo software for data analysis.

Cell population abundance
No sorting experiments were performed.

Gating strategy
Gating strategy for blood and lung are described in Supplementary Figure 2.

All samples were initially gated using forward scatter and time. For blood samples, forward and side scatter were used to identify live, single cell lymphocytes before gating for T cells and relevant subpopulations. For lung samples, CD45 and viability dye to identify events corresponding to live hemapoietic cells, after which forward and side scatter were used to identify lung lymphocytes before gating for T cells and relevant subpopulations.

☒ Tick this box to confirm that a figure exemplifying the gating strategy is provided in the Supplementary Information.
